# XIST lost induces ovarian cancer stem cells to acquire taxol resistance via a KMT2C-dependent way

**DOI:** 10.1186/s12935-020-01500-8

**Published:** 2020-09-04

**Authors:** Ruili Huang, Lijuan Zhu, Yali Zhang

**Affiliations:** Department of Gynaecology and Obstetrics, The First People’s Hospital of Shangqiu, Henan, No. 292 Kaixuan South Road, 476100 Shangqiu, Henan People’s Republic of China

**Keywords:** LncRNA XIST, KMT2C, Ovarian cancer, Tumor stem cells, Proliferation

## Abstract

**Background/aims:**

The expression levels of long non-coding RNA XIST are significantly associated with paclitaxel (Pac) sensitivity in ovarian cancer, but the mechanism of action remains unclear. Therefore, this experimental design was based on lncRNA XIST analysis to regulate the effect of XIST on the tumor stem cell and paclitaxel sensitivity in ovarian cancer.

**Methods:**

Sphere assay and fluorescence activated cell sorting (FACS) were used to determine the expression levels of XIST and sensitivity to paclitaxel treatment. The effect of the proliferation was detected by MTT assay. Target gene prediction and screening, luciferase reporter assays were used to validate downstream target genes for lncRNA XIS and KMT2C. The expression of KMT2C was detected by RT-qPCR and Western blotting. RT-qPCR was used to detect the expression of cancer stem cell-associated genes SOX2, OCT4 and Nanog. The tumor changes in mice were detected by in vivo experiments in nude mice.

**Results:**

There was an inverse correlation between the expression of XIST and cancer stem cell (CD44 + /CD24−) population. XIST promoted methylation of histone H3 methylation at lysine 4 by enhancing the stability of lysine (K)-specific methyltransferase 2C (KMT2C) mRNA. XIST acted on the stability of KMT2C mRNA by directly targeting miR-93-5p. Overexpression of miR-93-5p can reverse the XIST overexpression-induced KMT2C decrease and sphere number increase. Overexpression of KMT2C inhibited XIST silencing-induced proliferation of cancer stem cells, and KMT2C was able to mediate paclitaxel resistance induced by XIST in ovarian cancer. The study found that XIST can affect the expression of KMT2C in the ovarian cancer via targeting miR-93-5p.

**Conclusion:**

XIST promoted the sensitivity of ovarian cancer stem cells to paclitaxel in a KMT2C-dependent manner.

## Background

Ovarian cancer is one of the three major malignant tumors in female reproductive organs [1, 2]. The incidence rate is second only to cervical cancer and endometrial cancer, but its mortality rate ranks first among gynecological tumors [3]. Ovarian cancer has become a malignant tumor that seriously threatens women's health and life. Clinically, although chemotherapy has a positive effect on tumor control, it is still not clear about improving survival rate and reducing distant metastasis [4]. Therefore, it is particularly necessary to explore new targeted treatments for inhibiting tumor recurrence. In recent years, with the deepening of research on the mechanism of tumorigenesis and development, it has been found that the growth, metastasis and recurrence of malignant tumors are related to stem cells [5]. Tumor stem cells (TCCs) are abnormal tissues formed by the proliferation of tumor-forming TCCs. TCCs are closely related to the malignant biological behavior of tumors, and are the determinants of tumorigenesis, development, invasion, metastasis, and drug resistance [6]. The theory of cancer stem cells proposes a new elaboration for the origin of tumors, pointing out new targets for the treatment of tumors.

Long non-coding RNAs (lncRNAs) are a class of non-coding RNA molecules that are more than 200 nucleotides in length and do not encode proteins [7]. They are involved in many biological processes, such as participation in protein coding [8]. Most of the known lncRNAs are involved in many biological processes at the epigenetic, transcriptional, and post-transcriptional levels [9]. More importantly, the dysregulation of lncRNAs in cancer involves the progression of a range of diseases [10, 11]. In vitro and in vivo functional analysis have shown that lncRNAs are involved in a variety of cancer malignant behaviors such as unrestricted proliferation, metastasis, radiation therapy, and cancer stem cell phenotype [12, 13]. The X-chromosome inactivating specific transcript (XIST) gene is localized to the X-chromosome inactivation center (Xic) and encodes RNA longer than 200 bp [14]. Studies have demonstrated that lncRNA XIST plays a regulatory role in multiple tumors [15]. At present, the regulation mechanism of lncRNAs in paclitaxel resistance has not been reported.

In recent years, the role of miRNAs in the decline of chemotherapeutic drug sensitivity and drug resistance has attracted widespread attention [16]. Studies have shown that miRNAs can affect the sensitivity of tumor cells to chemotherapy drugs by regulating apoptosis regulators and drug transporters [17]. Studies have found that miR-93-5p is closely related to tumorigenesis [18]. The KMT2C gene has mutations or deletions in many tumors, including leukemia, liver cancer, pancreatic cancer, gastric cancer, cholangiocarcinoma, ovarian cancer, and bladder transitional cell carcinoma [19, 20]. In recent years, several studies have found that lncRNAs may play a biological role by regulating downstream target genes through miRNA adsorption processes [21]. Based on the above studies, it was hypothesized that lncRNA XIST may regulate the progression of ovarian cancer stem cells through miR-93-5p/KMT2C. The main purpose of this study was to investigate the mechanism of action of lncRNA XIST in the regulation of ovarian cancer stem cells, and to open a new way to reverse the drug resistance of paclitaxel in ovarian cancer.

## Materials and methods

### Sample collection

A total of 87 patients with ovarian cancer were confirmed by pathology, and surgical resection was performed at the First People’s Hospital of Shangqiu between 2014 and 2016. All patients did not receive chemotherapy or radiation before enrollment. The patient's ovary cancer tissues and adjacent normal tissues were collected. The clinical stage of the patient was assessed according to the International Union Against Cancer (UIAC) tumor-lymph node metastasis (TNM) system. The clinicopathological features of the patients were shown in Additional file 1: Table S1.

### Cell culture

SKOV3, ES-2, TOV21G and RMG-1 cell were purchased from the American Tissue Culture Collection. Taxol-resistant SKOV3/txr cell was obtained through Low dose sustained Taxol induction based on parental SKOV3 as reported in the previous research [22]. ES-2 was cultured in McCoy's SA medium supplemented with 10% FCS, while other ovarian cells were cultured in DMEM modified medium supplemented with 10% FCS.

### Cell transfection

For cell experiments, the plasmid based siRNA targeting for XIST and XIST overexpression vector were purchased from Shanghai Genepharma Co., Ltd. siRNA-XIST: 5′-AATGGAACGGGCTGAGTTTTAG-3′. MiR-93-5p mimics, miR-93-5p inhibitor and miRNA control were purchased from RiboBio Co., Ltd., Guangzhou, China. Briefly, 100 nM of miR-93-5p mimics or miR-93-5p inhibitor or 1000 ng plasmid were transfected with each 6-well plate for 48 h using Lipofectamine 2000 (Invitrogen, Waltham, MA, USA). For animal experiments, cells were stably transfected with XIST based on lentiviral, constructed by Hanyin Biotechnology Co., Ltd., Shanghai, China. After transfection, the DMEM was replaced with DMEM supplemented with puromycin (3 μg/ml) for excluding non-infected cells. Then, the selected cell were used in the subcutaneous xenograft.

### Dual luciferase reporter assay

To predict the target gene of XIST, we first performed a bioinformatics analysis via bioinformatic software targetscan 7.2 and starbase. MiR-93-5p was selected as the potential genes based on the RNAhybrid results. Then, the XIST sequence containing the predicted miR-93-5p binding site or mutation site was subcloned and inserted into the pmirGLO vector (Promega, Madison, WI, USA). After incubation, the recombinant plasmid or the empty pmirGLO vector (200 ng) and the luciferase reporter plasmid having the miR-93-5p consensus recognition site were co-transfected. Cells were co-transfected with these reporter plasmids and miR-93-5p inhibitors or mimetics, respectively. After 48 h of transfection, luciferase activity was measured using a dual luciferase assay system (Promega).

### RNA immunoprecipitation (RIP) assay

RIP experiments were performed using the Magna RIP RNA-Binding Protein Immunoprecipitation Kit (Millipore, Billerica, MA, USA) according to the manufacturer's instructions. Briefly, cells were lysed in RIP buffer after transfected with miR-93-5p mimics and followed with incubation with anti-Argonaute 2 (AGO2, SAB4200085, Millipore, USA) or anti-IgG antibody (R2655, Millipore, USA) at 4 °C overnight. The pellets were washed with PBS and resuspended in Tri Reagent (Sigma-Aldrich). Co-precipitated RNA was detected by reverse transcription PCR.

### Quantitative real-time PCR (qRT-PCR)

Total RNAs were extracted using TRIzol reagent (Invitrogen, Carlsbad, CA, USA). For miR-93-5p, the reverse transcription reaction was performed using TaqMan MicroRNA Reverse Transcription Kit (Applied Biosystems, Foster City, CA, USA). For lnc RNA XIST, the reverse transcription reaction was performed by a PrimeScript RT reagent kit (TaKaRa, Japan). These cDNA samples were amplified with the SYBR® Green PCR mix (Applied Biosystems) to detect the expression levels of XIST and miR-93-5p with the following the conditions: 95 °C for 10 min; 35 cycles of 95 °C for 15 s and 60 °C for 60 s. Glyceraldehyde-3-phosphate dehydrogenase (GAPDH) and U6 were used as internal control for mRNA and miRNA, respectively. The primer sequences were shown in Additional file 1: Table S1.

### Western blot analysis

The transfected cells were collected, total proteins were extracted, and the protein concentration was quantified using the BCA Protein As-say Kit. Membranes were incubated with primary antibody (anti-ABCB1, anti-ABCC1 and anti-ABCG2 (Novus Biologicals, USA), anti-PTEN (Abeam, USA), anti-p-Akt, anti-t-Akt, anti-p-mTOR and anti-t-mTOR (Cell Signaling Technology, USA) and anti-β-actin (Proteintech, USA) at 4 °C overnight. Then membranes were incubated with anti-rabbit/mouse IgG secondary antibody (Jackson, USA). Western blot analysis were performed as described in previous study [23].

### Sphere assay

SKOV3 cells were cultured in serum-free medium supplemented with 5 μg/mL insulin (Sigma, St. Louis, MO, USA), 20 ng/mL human recombinant epidermal growth factor (EGF, Invitrogen) and 10 ng/mL basic fibroblasts. Growth factors (bFGF; Invitrogen) and 0.4% bovine serum albumin (BSA, Invitrogen) were added in 6-hole UltraLow attachment plates (Corning, Corning, NY, USA) with a density of 10^4^/hole for 10 d. A sphere having a size of 20 μm or more was counted.

### Fluorescence activated cell sorting (FACS)

SKOV3 cells were resuspended in PBS and 5 × 10^5^ cells were taken for FACS analysis. Specific antibodies used for FACS analysis were PE-conjugated anti-human FITC-conjugated CD44 antibody, PE-conjugated CD24 antibody. Then cells were analyzed on a BD FACSCalibur after incubation in the dark.

### Cell proliferation assay

Cells at the logarithmic growth phase were trypsinized and adjusted to a cell density of 1 × 10 ^4^ mL^−1^. The cells were seeded in a 96-well plate. After the cells were attached, freshly prepared 5 g·L-1 MTT solution was added to each well. The culture was continued for 4 h, the supernatant was aspirated, and 150 μL of DMSO was added to each well. The absorbance was measured at a wavelength of 490 nm by a microplate reader (SAFAS XeniusXL, Ruixuan, Shanghai, China).

### Subcutaneous xenograft mouse model

Animal research was approved by the Institutional Animal Care and Use Committee of Nanjing Medical University. Thirty female nude mice (6–8 weeks old) were purchased from the Shanghai Experimental Animal Center of the Chinese Academy of Sciences. They were randomly divided into 6 groups (n = 5), and the corresponding drug treatment was given when the subcutaneous tumor volume was increased to about 100 mm^3^ (day 11 after inoculation). Paclitaxel injection of 20 mg/kg concentration was injected intraperitoneally every week. TOV21G with XIST overexpression, which were performed with stable transfection, were subcutaneously inoculated into nude mice. As a control group, TOV21G without XIST transfection were subcutaneously inoculated into nude mice. Tumor volume: V = (width^2^ × length) / 2. Mice were sacrificed on day 30 starting from the xenograft graft.

### Statistical analysis

The monitoring data were analyzed by SPSS19.0 statistical software. Data were shown as mean ± standard deviation (SD). Kaplan–Meier was used to evaluate 5-year tumor-free survival rate. Multi group data analysis was based on one-way ANOVA. LSD test was used for subsequent analysis. P < 0.05 indicated that the difference was significant.

## Results

The expression levels of XIST were correlated with cancer stem cell population and sensitivity to Taxol treatment.

**Fig. 1:**
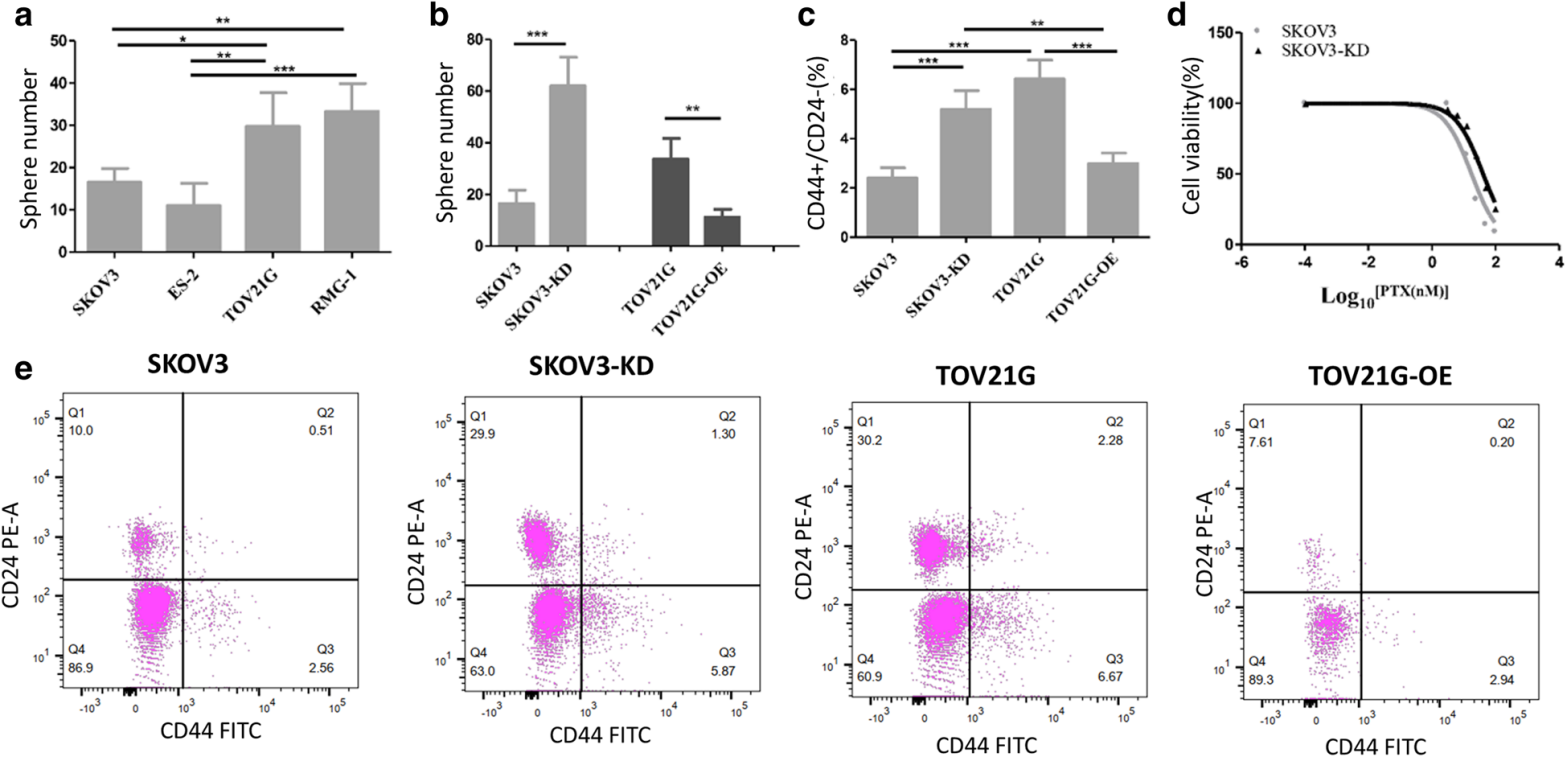
XIST expression levels correlated with cancer stem cell populations and sensitivity to paclitaxel treatment. **a** Ball formation efficiency (SFE) of different ovarian cancer cell lines. **b** XIST expression levels correlated with SFE of SKOV3 and TOV21G. **c** XIST expression levels correlated with the CD44 + / CD24− population in SKOV3 and TOV21G. **d** XIST regulated the sensitivity of SKOV3 and TOV21G to paclitaxel. *p < 0.05, **p < 0.01, ***p < 0.001

As shown in the Fig. 1a, the sphere number of SKOV3 was significantly lower than that of TOV21G (*p* < 0.01). On the contrary, the expression levels of XIST in SKOV3 were significantly higher than that of TOV21G (*p* < 0.01) (Additional file 2: Fig. S1). In order to explore the relation between the sphere formation efficiency (SFE) and the expression of XIST, XIST was knocked down in the SKOV3, labelled as SKOV3-KD, and overexpressed in TOV21G, respectively, labelled as TOV21G-OE (Fig. SB). Next, we conducted sphere assay and FACs assay in the four groups. As shown in Fig. 1b, the SFE of SKOV3-KD was significantly higher than that of parent SKOV3 (*p* < 0.01), whereas the SFE of TOV21G-OE was significantly lower than that of parent TOV21G (*p* < 0.01). The percentage of CD44 + / CD24− cells of SKOV3-KD was significantly higher than that of parental SKOV3 (*p* < 0.01), whereas the percentage of CD44 + / CD24− cells of TOV21G-OE was significantly lower than that of parental SKOV3 (*p* < 0.01) (Fig. 1c, e). In addition, the sensitivity of SKOV3 and SKOV3-KD to paclitaxel were examined by MTT assay. As shown in Fig. 1d, the IC50 value of SKOV3-KD was 2.5 times higher than SKOV3 (*p* < 0.01). We also measured the effect of knockdown of XIST on sensitivity of the taxol-resistant cell line SKOV3(Figure SC), and found that IC50 value of the knocked-down of XIST taxol-resistant SKOV3-KD was 2.7 times higher than the taxol-resistant SKOV3. These results indicated that the expression levels of XIST were correlated with cancer stem cell population and sensitivity to Taxol treatment.

### XIST upregulated KMT2C by stabilizing its mRNA

**Fig. 2:**
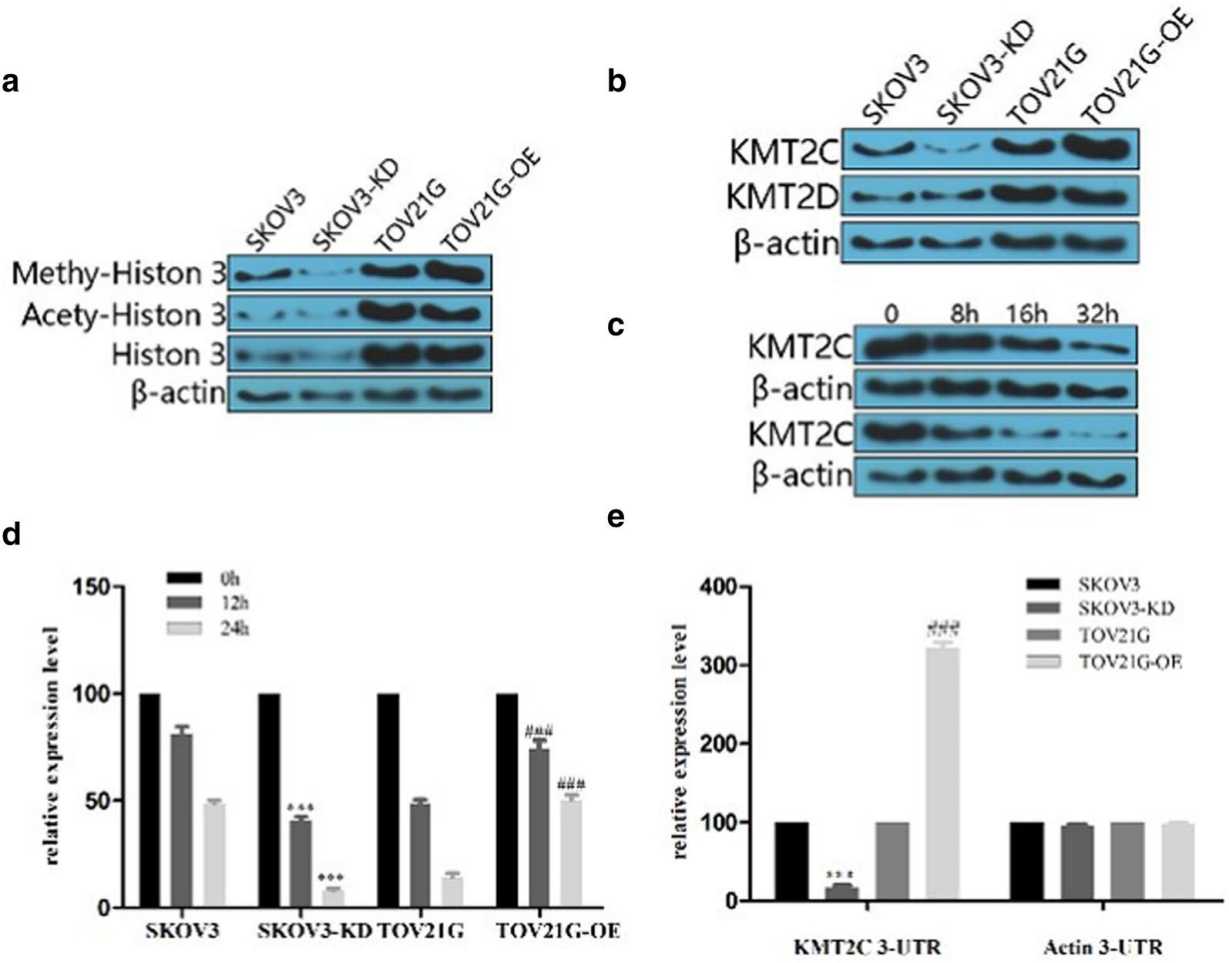
XIST up-regulated KMT2C expression by stabilizing its mRNA. **a** XIST expression was associated with methylation of histone 3. **b** XIST regulated KMT2C expression levels. **c** XIST did not regulate the stability of the KMT2C protein. **d** XIST increased the stability of KMT2C mRNA. **e** XIST regulated KMT2C mRNA stability by its 3-UTR. *: compared with SKOV3, ^#^: compared with TOV21G. *p < 0.05, **p < 0.01, ***p < 0.001; ^#^ p < 0.05, ^##^ p < 0.01, ^###^ p < 0.001

Next, we investigated the methylation-H3K4 and the expression of KMT2C and KMT2DAs in the four groups. As shown in Fig. 2a, the methylation of H3K4 in TOV21G-OE was significantly higher than TOV21G. Compared with the SKOV3, the methylation-H3K4 of SKOV3-KD was significantly reduced. KMT2C was significantly up-regulated in TOV21G-OE compared with that in parental TOV21G (Fig. 2b). The expression levels of KMT2C were significantly reduced in SKOV3-KD compared with that in parental SKOV3. However, the expression of KMT2D was not significantly altered. In addition, overexpression of XIST was unable to regulate the stability of the KMT2C protein, evidenced by gradual reduction of the expression levels of KMT2C in the TOV21G and TOV21G-OE group as the duration of action was extended (Fig. 2c). However, TOV21G-OE group showed more delayed degradation of KMT2C mRNA than TOV21G within 24 h, indicating that overexpression of XIST effectively inhibited the degradation of KMT2C mRNA (Fig. 2d). Furthermore, Luciferase experiments showed that the relative luciferase expression in TOV21G-OE was three times higher than that of the parental TOV21G, while the relative luciferase expression in SKOV3-KD was five times lower than that of the parental SKOV3. However, when KMT2C 3′-UTR was replaced by actin 3-UTR, there was no significant difference between SKOV3-KD and SKOV3 or TOV21G-OV and TOV21G (Fig. 2e), indicating that XIST can bind with 3′-UTR of KMT2C. Combined with that SKOV3 had the highest expression levels of XIST and TOV21G-OV had the lowest expression levels (Fig. SA), we can concluded that XIST could increase the expression levels of KMT2C by modulating its mRNA stability via targeting its 3′-UTR.

The expression of XIST was correlated with KMT2C and survival rate in patients with ovarian cancer.

**Fig. 3:**
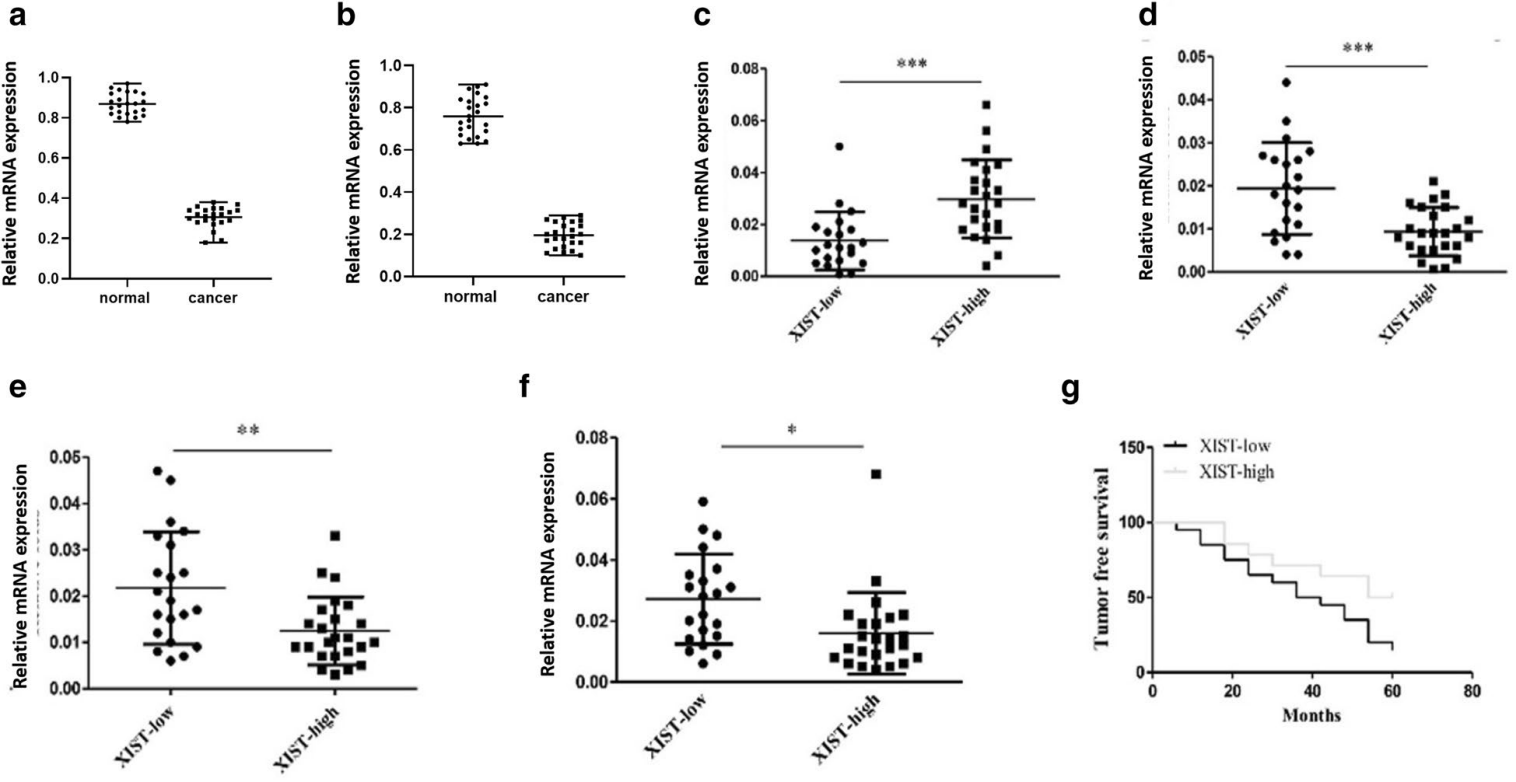
XIST correlated with KMT2C and cancer stem cell-associated gene expression in ovarian cancer tumor tissues. **a**. Relative mRNA expression of XIST in the normal tissues from the patients. **b** Relative mRNA expression of KMT2C in the normal tissues from the patients. **c** Relative mRNA expression of KMT2C in human ovarian tumor tissues. **d** Relative mRNA expression of SOX2 in human ovarian tumor tissues. **e** Relative mRNA expression of SOX2 in human ovarian tumor tissues. **f** Relative mRNA expression of and Nanog in human ovarian tumor tissues. **g** Five-year disease-free survival was assessed using the Kaplan–Meier method. *p < 0.05, **p < 0.01, ***p < 0.001

Next, the correlation between XIST and KMT2C and cancer stem cell-related genes in human ovarian tumor tissues was analyzed. We collected 87 samples and their adjacent normal ovarian tissues and measured the expression of XIST and KIMT2C. Cancer tissues showed a significant lower expression levels of XIST (Fig. 3a) and KIMT2C (Fig. 3b) than that in normal tissues (*p* < 0.01). The 87 samples were divided into XIST-high and XIST-low according to the expression levels of XIST. As shown in Fig. 3c, the expression levels of KMT2C were significantly higher in the XIST-high group than that in the XIST-low group (*p* < 0.01). In the XIST-high group, the expression levels of SOIST2, OCT4 and Nanog were significantly lower than those of the XIST-low group (*p* < 0.01) (Fig. 3d–f). In addition, the 5-year tumor-free survival rate of patients in the XIST high expression group was significantly higher than that in patients with low expression levels of XIST (*p* < 0.01) (Fig. 3g). We concluded that patients with high expression levels of XIST tended to show high expression levels of KMT2C and low expression levels of SOIST2, OCT4 and Nanog as well as high 5-year tumor-free survival rate.

### MiR-93-5p mediated XIST-induced KMT2C upregulation

**Fig. 4:**
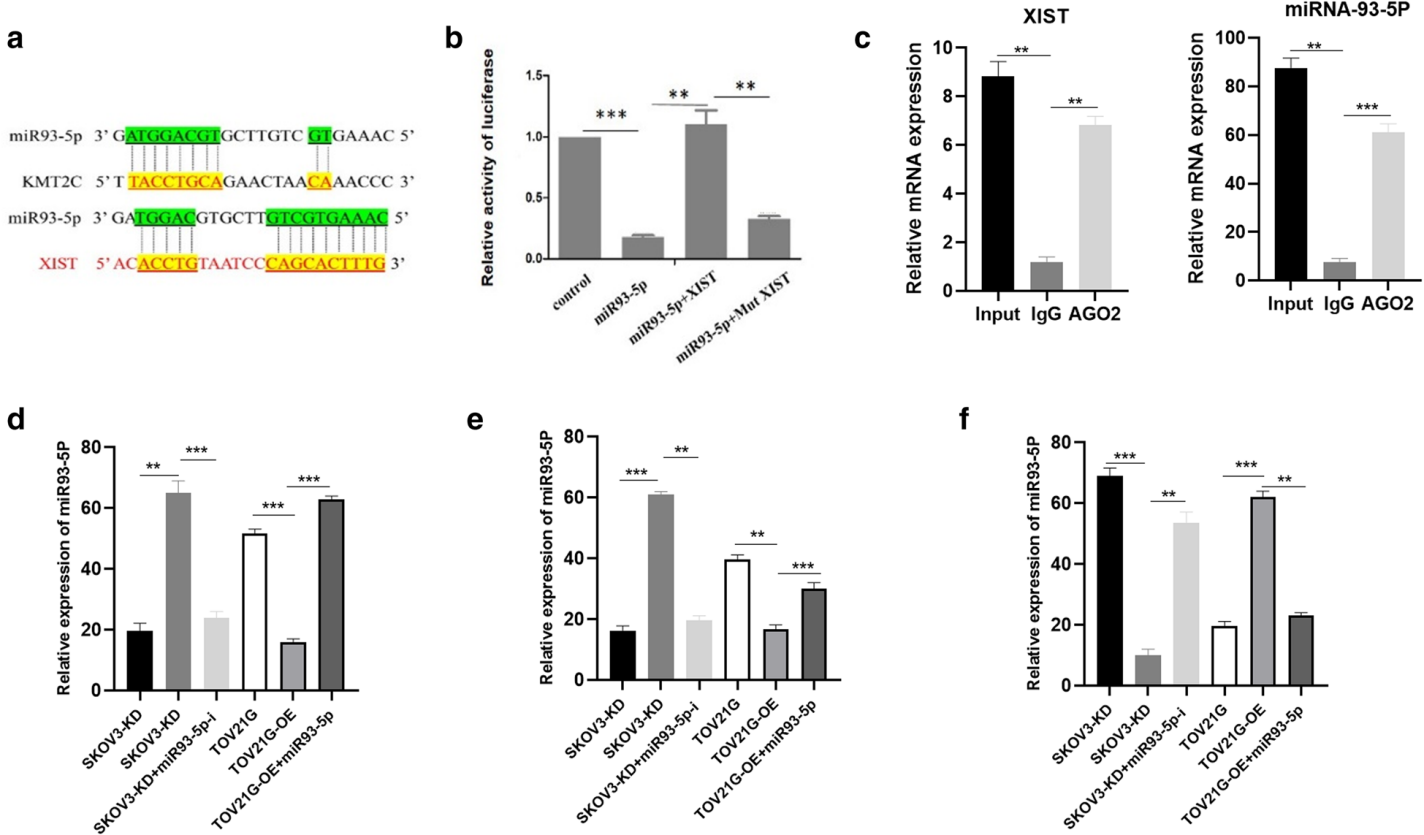
miR-93-5p mediated XIST-induced up-regulation of KMT2C. **a** Prediction of the binding site of miR-93-5p in XIST and KMT2C mRNA 3-UTR. **b** Luciferase reporter assay of XIST and miR-93-5p. **c** RNA immunoprecipitation assay of XIST and miRNA-93-5P. **d** Relative expression of miRNA-93-5P in each group. **e** Sphere number in each group. **f** Relative expression of KMT2C in each group. **p < 0.01, ***p < 0.001

It was predicted by bioinformatics that miRNA 93-5p was a potential target for XIST and KMT2C. The predicted binding sites of miRNA 93-5p sequence and 3′-UTRs of its target genes are shown in Fig. 4a. In order to confirm the direct relation between miRNA 93-5p and XIST, we conducted dual luciferase report assay. It revealed that the activity of luciferase reporter was significantly elevated by co-transfection of miRNA 93-5p and XIST, which was abrogated when the target site was mutated (Fig. 4b). RNA immunoprecipitation revealed that AGO2 antibody was able to pull down both endogenous XIST and miR-93-5P (Fig. 4c), further demonstrating that miR-93-5P was the target of XIST (Fig. 4c). Furthermore, miR-93-5P inhibitor significantly abolished the elevated SFE and reduced the expression levels of KMT2C induced by XIST knockdown in the SKOV2 (*p* < 0.01). In the meantime, the transfection of miR-93-5P mimics remarkably inverted the decreased SFE and increased KMT2C expression induced by overexpression of XIST in the SKOV2 (*p* < 0.01). These data indicated that miRNA 93-5p mediated XIST-induced KMT2C upregulation.

Overexpression of KMT2C impaired XIST lost-induced cancer stem cells and Taxol resistance in vitro*.*

**Fig. 5:**
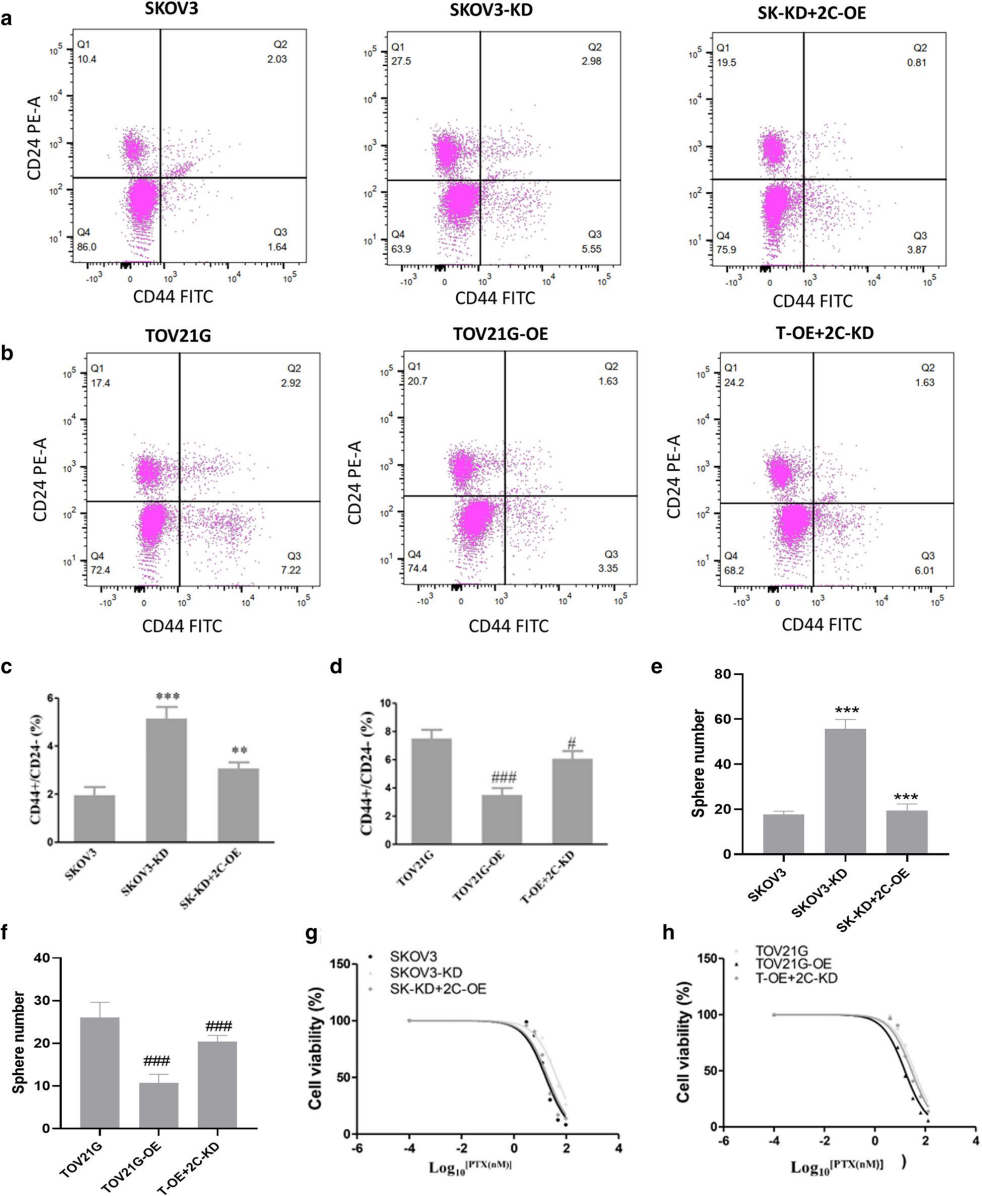
KMT2C mediated XIST silencing-induced cancer stem cells and in vitro paclitaxel resistance. **a**, **b** Effect of KMT2C on the number of CD44 +  CD24−population cells induced by XIST silencing. **c** KMT2C expression was associated with SFE in SKOV3 and TOV21G.**d** KMT2C was associated with SKOV3 and **e** TOV21G with paclitaxel sensitivity. *: compared with SKOV3; ^#^: compared with TOV21G. *p < 0.05, **p < 0.01, ***p < 0.001; ^#^ p < 0.05, ^##^ p < 0.01, ^###^ p < 0.001

To further confirm the role of KMT2C in the cancer stem cells and Taxol resistance, we respectively overexpressed XIST in the SKOV3-KD group, as well as knocked down XIST in the TOV21G-OE group, labelled as SK-KD + 2C-OE and T-OE + 2C-KD, respectively. As a result, the expression levels of KMT2C were greatly elevated in the SK-KD + 2C-OE group compared with SKOV3-KD (Fig. SD). The expression of KMT2C was obviously reduced in the T-OE + 2C-KD compared with TOV21G-OE (Fig. SE). The overexpression of KMT2C significantly decreased the up-regulation of the CD44 + /CD24 population in the SKOV3 cell induced by XIST knockdown (*p* < 0.01) (Fig. 5a, c). Furthermore, the reduction of KMT2C notably inverted the down-regulation of the CD44 + / CD24− population in TOV21G cell induced by XIST overexpression (*p* < 0.01) (Fig. 5b, d). Consistent with its role in the CD44 + / CD24− population, the knockdown of KMT2C significantly elevated the SFE of SKOV3 (P < 0.01), while the over expression of KMT2C significantly inhibited the SFE of TOV21G, indicating that KMT2C was directly related to the cancer stem cells (*p* < 0.01) (Fig. 5e, f). Furthermore, overexpression of KMT2C reduced the IC50 value in SKOV3 elevated by knockdown of XIST (*p* < 0.01) (Fig. 5g), while knockdown of KMT2C increased the IC50 value in TOV21G reduced by overexpression of XIST (*p* < 0.01) (Fig. 5h). These results demonstrated that overexpression of KMT2C impaired XIST lost-induced cancer stem cells and Taxol resistance in vitro.

Overexpression of XIST sensitized ovarian cancer cells to Taxol treatment by decreasing cancer stem cells in vivo*.*

**Fig. 6:**
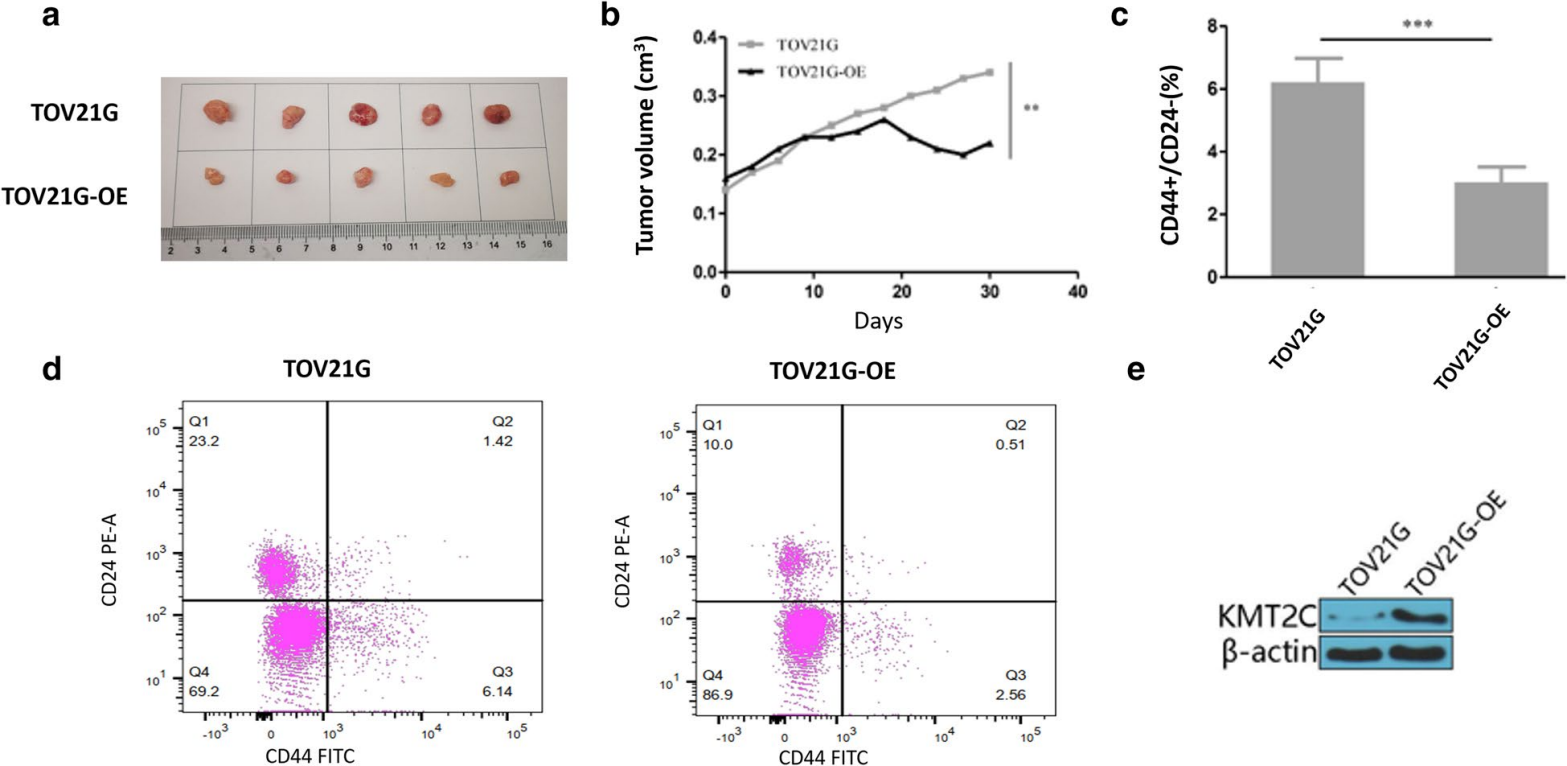
XIST overexpression reduced cancer stem cells in vivo and thereby increased the sensitivity of ovarian cancer cells to Taxol treatment. **a** XIST overexpression impaired tumor volume in ovarian cancer cells. **b** XIST overexpression improved the role of paclitaxel in the body. **c** XIST correlated with the percentage of CD44 +  CD24−population in tumors. **d** KMT2C protein expression in tumors of TOV21G-OE and parental TOV21G.*p < 0.05, **p < 0.01

Finally, the effect of overexpression of XIST on the sensitivity of tumor cells to paclitaxel was examined in vivo by subcutaneous xenograft of XIST overexpressed TOV21G in mice. The mice were treated with PTX once the diameter of tumors reached 3 mm. We collected cancer tissues after subcutaneous xenograft 30 days (Fig. 6a). As expected, tumors from TOV21G-OE almost stopped growing, while control tumors still grew, indicating that overexpression of XIST drastically hampered tumor growth compared to control group (*p* < 0.01) (Fig. 6b). Furthermore, as shown in Fig. 6c, d, the number of CD44 + /CD24−population cells in mice grafted with XIST overexpressed TOV21G was significantly reduced compared with mice grafted with TOV21G (*p* < 0.01). Besides, the expression of KMT2C was significantly elevated in tumors from TOV21G-OE (*p* < 0.01) (Fig. 6e). These results indicated that overexpression of XIST increased the sensitivity of tumor cells to paclitaxel by inhibiting cancer stem cells and upregulating KMT2C.

## Discussion

Ovarian cancer is the most common malignant tumor of the female reproductive system, which seriously threatens women's life and health [24]. The important methods of treatment of ovarian cancer is surgical therapy, combined with chemotherapy via paclitaxel (Pac) and platinum [25, 26]. Pac is a new anticancer drug extracted from Taxusbrevifolia. Clinical studies in the United States have shown that Pac has outstanding efficacy in ovarian cancer and breast cancer [27]. As a first-line chemotherapeutic drug, Pac is mainly used to treat ovarian cancer. However, the tumor cell's resistance to Pac leads to tumor recurrence, rendering just 40% overall five-year survival rate in ovarian cancer patients [28].

Studies have shown that lncRNAs play important roles in many biological activities such as epigenetic regulation, cell cycle regulation and cell differentiation regulation [10]. Studies have shown that the abnormal expression of lncRNA is closely related to the occurrence, development, invasion and metastasis of ovarian cancer [29, 30]. Studies have found that the expression of lncRNA NEAT1 is abnormally elevated in breast and ovarian cancer, and interference with the expression of lncRNA NEAT1 in vitro inhibits the proliferation of ovarian cancer cells [31]. LncRNAs have been reported to be involved in tumorigenesis and drug sensitivity and drug resistance [32]. For example, studies have found that overexpression of lncRNA UCA1 in ovarian cancer cell line SKOV3 increases cellular cisplatin resistance, the mechanism of which may involve overexpression of RPK1 and subsequent dysregulation of the apoptotic pathway related protein [33]. Studies have confirmed that lncRNA XIST plays a regulatory role in multiple tumors [34, 35]. Our study found that SKOV3, which had highest XIST expression, showed significantly lower SFE than TOV21G that had lowest XIST expression. The percentage of SFE and CD44 + / CD24− cells of SKOV3-KD was significantly higher than that of parental SKOV3, while the result of TOV21G-OE was reversed. Moreover, XIST knockdown reduced IC50 value on SKOV3 and taxol-resistant cell line SKOV3, indicating that the expression of XIST was correlated with cancer stem cell population and sensitivity to Taxol treatment.

A growing body of evidence supports the important role of epigenetic regulation in the induction of cancer stem cells, including DNA methylation and histone modifications [36, 37].It has been demonstrated that XIST can act through epigenetic modifications [38]. In recent years, with the advancement of research methods such as high-throughput sequencing, the role of KMT2C in many solid tumors has become increasingly prominent [39]. KMT2C has a high mutation rate in the sequencing data of cancer specimens provided by different research institutions [40]. This study found that XIST increased KMT2C expression by regulating mRNA stability. The expression of KMT2C was significantly higher in the XIST high expression group than in the XIST low expression group. The expression of cancer stem cell-associated genes SOIST2, OCT4, and Nanog was significantly decreased in the XIST high expression patients. And the 5-year tumor-free survival rate of patients in the XIST high expression group was significantly increased. In addition, this study found that KMT2C overexpression inhibited XIST silencing-induced cancer stem cell and paclitaxel resistance in vitro. Furthermore, in vivo studies further confirmed that XIST overexpression increased the sensitivity of tumor cells to paclitaxel by down-regulating cancer stem cells. Thus, we can conclude that the regulation effect of XIST in Ovarian cancer stem cells and paclitaxel resistance depend on KMT2C.

In recent years, the research of miRNAs in tumor biological function is a hot spot. It not only regulates the normal metabolism of cells but also participates in the formation of tumors [41]. It can also reverse the sensitivity of tumor cells to chemotherapy drugs [42, 43]. Studies have found that miR-21-3p can target the regulation of NAV3 gene to reduce the sensitivity of ovarian cancer cells to paclitaxel [44]. Different miRNAs can play different biological roles by targeting different genes. Our research demonstrated that miRNA 93-5p was a potential target for XIST and KMT2C. Co-transfection with wild-type XIST reversed the expression level of luciferase activity in the miRNA93-5p overexpression group. Furthermore, overexpression of miRNA 93-5p can reverse the effect induced by overexpression of XIST. We concluded that miR-93-5p can mediate XIST-induced up-regulation of KMT2C expression.

## Conclusion

Overexpression of XIST can enhance the anti-cancer sensitivity of paclitaxel to ovarian cancer cells, and its effect may be related to the up-regulation of KMT2C. The results of this study suggested that miR-93-5p/XIST/ KMT2C signal axis can provide new potential therapeutic target and may play an important role in the treatment of ovarian cancer in the future.

## Supplementary information

**Additional file 1.** Additional tables.

**Additional file 2: Figure S1.** (A) Relative expression level of XIST in four cell lines. (B) Relative expression level of XIST in SKOV3, SKOV3-KD, TOV21G, TOV21G-OE. (C) WB assay in SKOV3, SKOV3-KD and SKOV3-KD+2C OE. (D) WB assay in TOV21G, TOV21G-OE and TOV21G-OE+2C KD. *: compared with SKOV3, #: compared with TOV21G. *p < 0.05, **p < 0.01, ***p < 0.001; ^#^p < 0.05, ^##^p < 0.01, ^###^p <0.001.

## Data Availability

The analyzed data sets generated during the study are available from the corresponding author on reasonable request.

## Abbreviations

Pac: Paclitaxel
TCCs: Tumor stem cells
XIST: X-chromosome inactivating specific transcript
FACS: Fluorescence activated cell sorting
KMT2C: Lysine (K)-specific methyltransferase 2C
SFE: Sphere formation efficiency
RIP: RNA immunoprecipitation
LncRNA: Long non-coding RNA

## References

[1] Holschneider CH, Berek JS (2015). Ovarian cancer: Epidemiology, biology, and prognostic factors. Semin Surg Oncol.

[2] Antoniou A, Pharoah PDP, Narod S, Risch HA, Eyfjord JE, Hopper JL, Borg Å (2003). Average risks of breast and ovarian cancer associated with BRCA1 or BRCA2 mutations detected in case series unselected for family history: a combined analysis of 22 studies. Am J Hum Genet.

[3] Pinsky P, Miller A, Kramer B, Church T, Reding D, Prorok P, Hayes R (2007). Evidence of a healthy volunteer effect in the prostate, lung, colorectal, and ovarian cancer screening trial. Am J Epidemiol.

[4] Agarwal R, Kaye SB (2003). Ovarian cancer: strategies for overcoming resistance to chemotherapy. Nat Rev Cancer.

[5] Jordan CT, Guzman ML, Noble M (2006). Cancer stem cells. N Engl J Med.

[6] Wu H, Zhao G, Zu H, Wang JHC, Wang QM (2015). Aging-related viscoelasticity variation of tendon stem cells (TSCs) characterized by quartz thickness shear mode (TSM) resonators. Sensors & Actuators B Chemical.

[7] Xu M, He Z, Ling L, Yang D, Liu G (2017). Expression profiles analysis of long non-coding RNAs identified novel lncRNA biomarkers with predictive value in outcome of cutaneous melanoma. Oncotarget.

[8] Goyal N, Kesharwani D, Datta M (2018). Lnc-ing non-coding RNAs with metabolism and diabetes: roles of lncRNAs. Cell Mol Life Sci.

[9] Richards EJ, Zhang G, Li ZP, Permuthwey J, Challa S, Li Y, Coppola D (2015). Long non-coding RNAs regulated by TGFβ: lncRNA-HIT mediated TGFβ-induced epithelial to mesenchymal transition in mammary epithelia. J Biol Chem.

[10] Yang G, Lu X, Yuan L (2014). LncRNA: A link between RNA and cancer. Biochem Biophys Acta.

[11] Xiang JF, Yin QF, Chen T, Zhang Y, Zhang XO, Wu Z, Lu X (2014). Human colorectal cancer-specific CCAT1-L lncRNA regulates long-range chromatin interactions at the MYC locus. Cell Res.

[12] Li H, Yu B, Li J, Su L, Yan M, Zhu Z, Liu B (2014). Overexpression of lncRNA H19 enhances carcinogenesis and metastasis of gastric cancer. Oncotarget.

[13] Yang Y, Li H, Hou S, Hu B, Liu J, Wang J (2013). The noncoding RNA expression profile and the effect of lncRNA AK126698 on cisplatin resistance in non-small-cell lung cancer cell. PLoS ONE.

[14] Engreitz JM, Pandyajones A, Mcdonel P, Shishkin A, Sirokman K, Surka C, Lander ES (2013). The Xist lncRNA exploits three-dimensional genome architecture to spread across the X-chromosome. Science.

[15] Du P, Zhao H, Peng R, Liu Q, Yuan J, Peng G, Liao Y (2017). LncRNA-XIST interacts withmiR-29cto modulate the chemoresistance of glioma cell to TMZ through DNA mismatch repair pathway. Biosci Rep.

[16] Smirnova L, Gräfe A, Seiler A, Schumacher S, Nitsch R, Wulczyn FG (2015). Regulation of miRNA expression during neural cell specification. Eur J Neurosci.

[17] Shimono Y, Zabala M, Cho RW, Lobo N, Dalerba P, Qian D, Chiao E (2009). Downregulation of miRNA-200c links breast cancer stem cells with normal stem cells. Cell.

[18] Fabbri E, Montagner G, Bianchi N, Finotti A, Borgatti M, Lampronti I, Gambari R (2016). MicroRNA miR-93-5p regulates expression of IL-8 and VEGF in neuroblastoma SK-N-AS cells. Oncol Rep.

[19] Koemans TS, Kleefstra T, Chubak MC, Stone MH, Reijnders M, De SM, Bok LA (2017). Functional convergence of histone methyltransferases EHMT1 and KMT2C involved in intellectual disability and autism spectrum disorder. PLoS Genet.

[20] Chiappetta C, Puggioni C, Carletti R (2018). The nuclear-cytoplasmic trafficking of a chromatin-modifying and remodelling protein (KMT2C), in osteosarcoma. Oncotarget.

[21] Saakshi J, Deeksha B, Kumar LM, Sridhar S, Vinod S (2013). Systematic transcriptome wide analysis of lncRNA-miRNA interactions. PLoS ONE.

[22] Sun NK, Huang SL, Chang TC, Chao CC (2018). TLR4 and NFκB signaling is critical for taxol resistance in ovarian carcinoma cells. Cellular physiology..

[23] Dalmau J, Furneaux HM, Gralla RJ, Kris MG, Posner JB (2010). Detection of the anti-Hu antibody in the serum of patients with small cell lung cancer–a quantitative western blot analysis. Ann Neurol.

[24] Piver MS, Jishi MF, Tsukada Y, Nava G (2015). Primary peritoneal carcinoma after prophylactic oophorectomy in women with a family history of ovarian cancer. A report of the Gilda Radner Familial Ovarian Cancer Registry. Cancer..

[25] Chang SJ, Bristow RE (2012). Evolution of surgical treatment paradigms for advanced-stage ovarian cancer: redefining 'optimal' residual disease. Gynecol Oncol.

[26] Kumpulainen S, Kuoppala T, Leminen A, Penttinen J, Puistola U, Pukkala E, Grénman S (2006). Surgical treatment of ovarian cancer in different hospital categories—a prospective nation-wide study in Finland. Eur J Cancer.

[27] Kupryjanczyk J, Kraszewska E, Ziolkowskaseta I, Madry R, Timorek A, Markowska J, Bidzinski M (2008). TP53 status and taxane-platinum versus platinum-based therapy in ovarian cancer patients: A non-randomized retrospective study. Bmc Cancer.

[28] Xiao K, Luo J, Fowler W, Li Y, Lee J, Wang L, Lam KS (2009). A self-assembling nanoparticle for paclitaxel delivery in ovarian cancer. Biomaterials.

[29] Zhou M, Wang X, Shi H, Cheng L, Wang Z, Zhao H, Sun J (2016). Characterization of long non-coding RNA-associated ceRNA network to reveal potential prognostic lncRNA biomarkers in human ovarian cancer. Oncotarget.

[30] Gao Y, Meng H, Liu S, Hu J, Zhang Y, Jiao T, Yao L (2015). LncRNA-HOST2 regulates cell biological behaviors in epithelial ovarian cancer through a mechanism involving microRNA let-7b. Hum Mol Genet.

[31] Chai Y, Liu J, Zhang Z, Liu L (2016). HuR-regulated lncRNA NEAT1 stability in tumorigenesis and progression of ovarian cancer. Cancer Med.

[32] Fang Q, Chen XY, Zhi XT (2016). Long non-coding RNA (LncRNA) urothelial carcinoma associated 1 (UCA1) increases multi-drug resistance of gastric cancer via downregulating miR-27b. Med Sci Monit Int Med J Exp Clin Res.

[33] Wang H, Guan Z, He K, Qian J, Cao J, Teng L (2017). LncRNA UCA1 in anti-cancer drug resistance. Oncotarget.

[34] Zhu H, Zheng T, Yu J, Zhou L, Wang L (2018). LncRNA XIST accelerates cervical cancer progression via upregulating Fus through competitively binding with miR-200a. Biomed Pharmacother.

[35] Xu R, Zhu X, Chen F, Huang C, Ai K, Wu H, Zhao X (2018). LncRNA XIST/miR-200c regulates the stemness properties and tumourigenicity of human bladder cancer stem cell-like cells. Cancer Cell Int.

[36] Kulis M, Esteller M (2010). DNA methylation and cancer. Adv Genet.

[37] Esteller M (2007). Cancer epigenomics: DNA methylomes and histone-modification maps. Nat Rev Genet.

[38] Zhou T, Qin G, Yang L, Xiang D, Li S (2017). LncRNA XIST regulates myocardial infarction by targeting miR-130a-3p. J Cell Physiol.

[39] Sato K, Akimoto K (2016). Expression levels of KMT2C and SLC20A1 identified by information-theoretical analysis are powerful prognostic biomarkers in estrogen receptor-positive breast cancer. Clinical Breast Cancer.

[40] Gala K, Li Q, Sinha A, Razavi P, Dorso M, Sanchezvega F, Berger M (2018). KMT2C mediates the estrogen dependence of breast cancer through regulation of ERα enhancer function. Oncogene.

[41] Croce CM, Calin GA (2005). miRNAs cancer, and stem cell division. Cell..

[42] Chen S, Jiao JW, Sun KX, Zong ZH, Zhao Y (2015). MicroRNA-133b targets glutathione S-transferase π expression to increase ovarian cancer cell sensitivity to chemotherapy drugs. Drug Des Dev Ther..

[43] Mei Y, Gao C, Wang K, Cui L, Li W, Zhao X, Ding W (2014). Effect of microRNA-210 on prognosis and response to chemotherapeutic drugs in pediatric acute lymphoblastic leukemia. Cancer Sci.

[44] Pink RC, Samuel P, Massa D, Caley DP, Brooks SA, Carter DR (2015). The passenger strand, miR-21-3p, plays a role in mediating cisplatin resistance in ovarian cancer cells. Gynecol Oncol.

