# Supplementary figures and images for: XIST lost induces ovarian cancer stem cells to acquire taxol resistance via a KMT2C-dependent way
Source: Cancer Cell Int. 2020 Sep 4;20:436. doi: 10.1186/s12935-020-01500-8 (PMC7487955; doi:10.1186/s12935-020-01500-8)

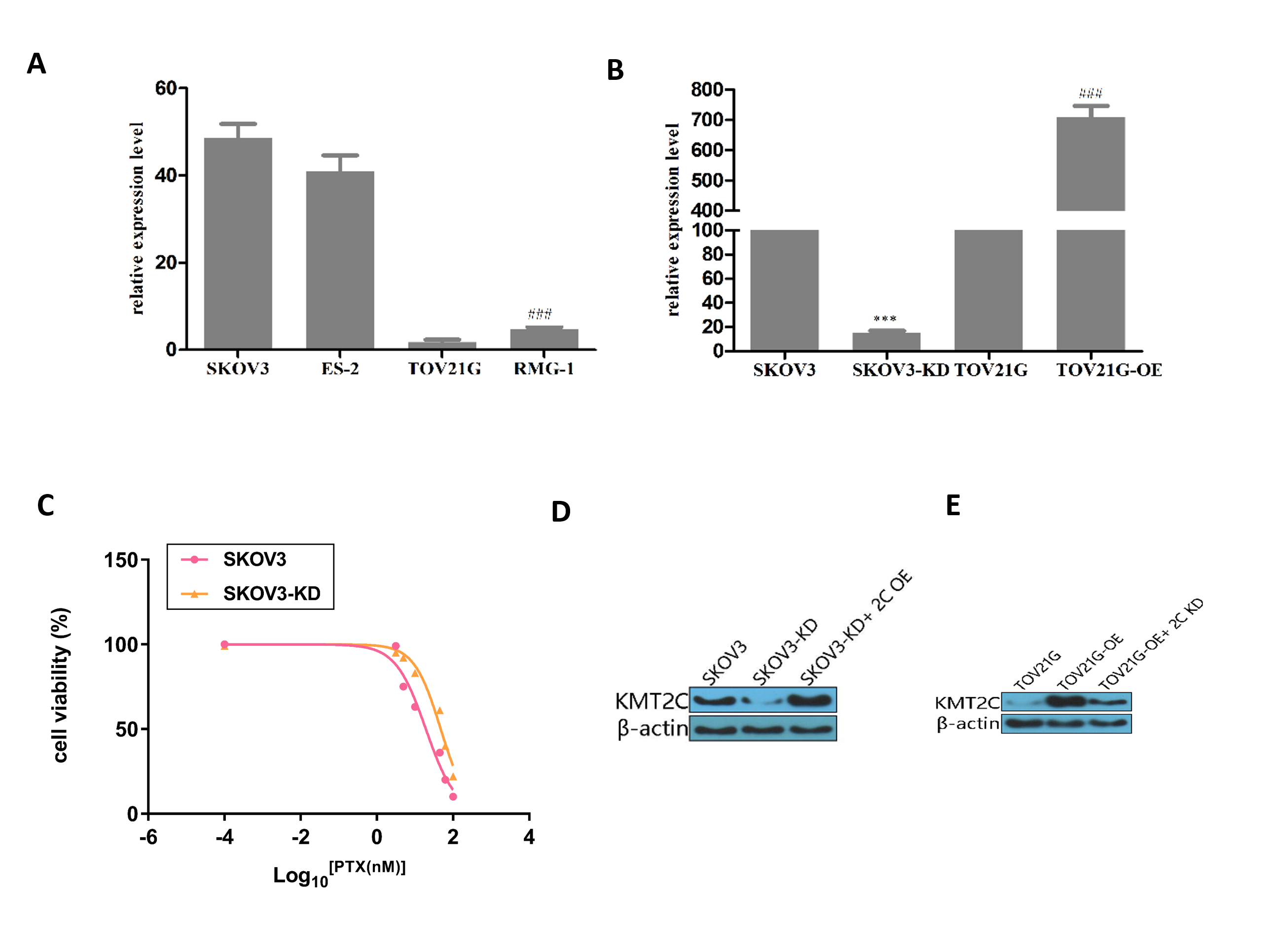

Supplement: Supplementary file 1 — Additional file 1. Additional tables. [file 12935_2020_1500_MOESM1_ESM.tif]
