# Supplementary material for: XIST lost induces ovarian cancer stem cells to acquire taxol resistance via a KMT2C-dependent way
Source: Cancer Cell Int. 2020 Sep 4;20:436. doi: 10.1186/s12935-020-01500-8 (PMC7487955; doi:10.1186/s12935-020-01500-8)
Supplement: Supplementary file 2 — Additional file 2: Figure S1. (A) Relative expression level of XIST in four cell lines. (B) Relative expression level of XIST in SKOV3, SKOV3-KD, TOV21G, TOV21G-OE. (C) WB assay in SKOV3, SKOV3-KD and SKOV3-KD+2C OE. (D) WB assay in TOV21G, TOV21G-OE and TOV21G-OE+2C KD. *: compared with SKOV3, #: compared with TOV21G. *p < 0.05, **p < 0.01, ***p < 0.001; #p < 0.05, ##p < 0.01, ###p <0.001. [file 12935_2020_1500_MOESM2_ESM.docx]

| SKOV3 | | |
| --- | --- | --- |
| Marker | Allele 1 | Allele 2 |
| SF1PO | 11 | 11 |
| 2S1338 | 18 | 23 |
| 3S1358 | 14 | 14 |
| 5S818 | 11 | 11 |
| D7S820 | 13 | 14 |
| D8S1179 | 14 | 15 |
| melogenin | X | X |
| D13S317 | 8 | 11 |
| D16S539 | 12 | 12 |
| D18S51 | 16 | 17 |
| D19S433 | 14 | 14.2 |
| D21S11 | 30 | 31.2 |
| GA | 24 | 25 |
| Penta D | 12 | 13 |
| Penta E | 5 | 13 |
| TH01 | 9 | 9.3 |
| TPOX | 8 | 11 |
| vWA | 17 | 18 |

**Table 1：STR spots of skov3 and genotyping**

**Table 2： STR spots of TOV-21G and genotyping**

| TOV-21G | | |
| --- | --- | --- |
| Marker | Allele 1 | Allele 2 |
| CSF1PO | 13 | 15 |
| D2S1338 | 17 | 17 |
| D3S1358 | 14 | 16 |
| D5S818 | 12 | 13 |
| D7S820 | 12 | 12 |
| D8S1179 | 13 | 16 |
| Amelogenin | X | X |
| D13S317 | 11 | 12 |
| D16S539 | 10 | 12 |
| D18S51 | 12 | 16 |
| D19S433 | 14 | 16.2 |
| D21S11 | 28 | 34.2 |
| FGA | 20 | 23 |
| Penta D | 9 | 9 |
| Penta E | 17 | 19 |
| TH01 | 7 | 9.3 |
| TPOX | 8 | 11 |
| vWA | 17 | 17 |

**Table 3： STR spots of RMG-1 and genotyping**

| RMG-1 | | |
| --- | --- | --- |
| Marker | Allele 1 | Allele 2 |
| CSF1PO | 10 | 10 |
| D3S1358 | 15 | 16 |
| D5S818 | 12 | 12 |
| D7S820 | 11 | 11 |
| D8S1179 | 15 | 16 |
| Amelogenin | X | X |
| D13S317 | 8 | 12 |
| D16S539 | 9 | 10 |
| D18S51 | 13 | 15 |
| D21S11 | 29 | 30 |
| FGA | 25 | 26 |
| Penta D | 9 | 10 |
| Penta E | 15 | 16 |
| TH01 | 6 | 7 |
| TPOX | 11 | 11 |
| vWA | 17 | 18 |

**Table 4： STR spots of ES-2 and genotyping**

| ES-2 | | |
| --- | --- | --- |
| Marker | Allele 1 | Allele 2 |
| D5s818 | 11 | 13 |
| D13S317 | 11 | 11 |
| D7S820 | 11 | 11 |
| D16S539 | 11 | 13 |
| VWA | 16 | 17 |
| TH01 | 9.3 | 9.3 |
| Amelogenin | X | X |
| TPOX | 8 | 12 |
| FGA | 21 | 21 |
| Penta D | 8 | 13 |
| Penta E | 13 | 16 |
| D2S1338 | 17 | 23 |
| D3S1358 | 15 | 18 |
| D8S1179 | 14 | 14 |
| D18S51 | 13 | 15 |
| D19S433 | 15 | 15.2 |
| D21S11 | 32.2 | 33.2 |
| CSF1PO | 10 | 15 |
